# Management of Gamma-Butyrolactone Dependence with Assisted Self-Administration of GBL

**DOI:** 10.1155/2014/485178

**Published:** 2014-06-26

**Authors:** Rafael Meyer, Josef Jenewein, Soenke Boettger

**Affiliations:** Department of Consultation-Liaison Psychiatry, University Hospital Zurich, Ramistraße 100, 8091 Zurich, Switzerland

## Abstract

Gamma-hydroxybutyric acid (GHB) and its liquid precursor gamma-butyrolactone (GBL) have become increasingly popular beyond the clubbing culture resulting in daily consumption and dependence in the broader population. This case report illustrates the challenges of managing GHB-withdrawal and a possibly superior future approach of its management by titration and tapering of the addictive agent.

## 1. Case Report

Mrs. M. is a 36-year-old Caucasian female with severe GBL-dependence, chronic kidney disease stage V on hemodialysis, and past alcohol, cocaine, amphetamine, and cannabis abuse. The patient is very well known from numerous medical and psychiatric hospitalizations. Once again, Mrs. M. presented to the emergency room of the University Hospital Zurich in GHB-withdrawal with subsequent delirium.

As she missed her hemodialysis when intoxicated, the patient was urgently hemodialyzed with no improvement in mental status. At that point, her mental status was severely impaired. She was lying on a stretcher, restless and unable to cooperate, grasping for two small brown glass bottles in her bag. Her consciousness was clouded, attention, concentration, and memory were severely impaired, and she was not able to produce a single meaningful sentence. Her thought process was incoherent as well as impoverished and no evidence for hallucinations or delusions existed. Her psychomotor behavior was restless and lacked goal direction, her affect was flat, impulsivity was nonexistent, and judgment and insight were severely impaired.

Laboratory studies revealed a mild known anemia; the hemoglobin was 114 g/L and hematocrit was 31.4% and mean corpuscular hemoglobin (MCH) as well as mean corpuscular hemoglobin concentration (MCHC) was increased. Electrolytes, liver function tests, and the remaining laboratory results were within normal limits. The EKG revealed a previously documented unspecific S-T elevation, and the urine toxicology tested only positive for GHB.

Mrs. M. was very well known to the hospital from previous admissions in GBL-withdrawal and associated delirium. She was managed and detoxified several times with oral and intravenous benzodiazepines. In those instances, the management course was challenging and complicated, with severe behavioral disturbances requiring intensive personal assistance in order to avert adverse outcomes. The withdrawal syndrome and delirium responded insufficiently due to the differences in pharmacodynamics between GHB and benzodiazepines. Even more, with oral and intravenous detoxification the hospitalization lasted on average more than a week resulting in significant health care costs.

As the patient obviously attempted to reach for her GBL-bottles, she was assisted in self-administration of GBL. The patient was aided in administration of 2–4 mL every 5 minutes, totaling approximately 20 mL over the next half hour. In this process her mental status cleared, the disturbance of consciousness remitted, orientation and cognition recovered, thought process and psychomotor behavior normalized, and decisional capacity was restored. Then she admitted to consumption of 40 mL GBL prior to hemodialysis. Despite recommendations to consider further detoxification and rehabilitation, the patient decided to leave the hospital against medical advice and since then has not presented again over the next weeks.

Among the admissions for GHB-withdrawal, the self-administration and titration of GBL presented the most efficient and cost effective approach.

## 2. Literature Review of GHB-Pharmacology, GHB-Dependence, GHB-Withdrawal, and GHB-Management

Initially GHB has been developed as an anesthetic and developed into a popular psychoactive drug in the clubbing scene [1]. Summarized from extensive reviews [2–4], GBL, a precursor of GHB, is primarily used as a solvent in the pharmaceutical industry and converted to GHB following hydrolysis by 1–4 lactonase. The plasma half-life of GBL is short, usually less than one minute. The time to peak serum levels ranges from 36 to 57 minutes, the elimination half-life between 30 and 52 minutes.

At least two distinct binding sites have been identified, the GHB-receptor and GABA_B_-receptor. The agonistic effect at the GHB-receptor is excitatory and the weak antagonistic effect at the GABA_B_-receptor is inhibitory. Activation of both receptors—GHB and GABA_B_—causes the addictive properties. The activation of the GHB-receptor causes the release of glutamate. The release of dopamine is biphasic: low doses stimulate dopamine release via the GHB-receptor, whereas higher concentrations then inhibit dopamine release via the GABA_B_-receptor and, eventually, after an initial phase of inhibition, dopamine release is again increased via the GHB-receptor.

More recently, in addition to the action at the low-affinity metabotropic GABA_B_-receptor, a high affinity binding site at the inotropic *α* *β* *δ*-GABA_A_-receptor site has been identified. However, the precise role of this high-affinity binding site still remains elusive [5, 6].

Addiction occurs when prolonged and repeated GHB use disrupts the balance of brain transmitters and circuits controlling reward, memory, and cognition leading to compulsive use. The duration of clinical effects is dose-dependent and ranges from 2.5 to 4 hours. The early GHB-withdrawal syndrome resembles the alcohol withdrawal syndrome which is associated with autonomic instability, tremor, restlessness, anxiety, and sleeping disorders. GHB-withdrawal usually lasts 3 to 21 days. Severe withdrawal syndromes can also produce acute delirium requiring hospitalization, often leading to intensive care management and possible fatal outcome. Important differential diagnoses for GHB-withdrawal syndrome include alcohol or benzodiazepine withdrawal, delirium caused by somatic conditions, neuroleptic malignant syndrome, and serotonin syndrome [3, 4]. The mainstay of management of the GHB-withdrawal syndrome remains the administration of benzodiazepines. Generally high doses are required. In contrast to GHB acting on the GABA_B_-receptor, benzodiazepines are indirect GABA_A_-agonists explaining the requirement for high doses of benzodiazepines [7]. To date, one study exists managing GHB-withdrawal with the administration, titration, and tapering of GHB itself [8].

## 3. Discussion

The case of Mrs. M. illustrates the challenges of managing GHB-withdrawal and potentially life-threatening complications such as rhabdomyolysis, seizures, bradycardia, and cardiac arrest. Due to the different pharmacodynamic mechanisms involving the GABA_B_-receptor in the case of GHB and the GABA_A_-receptor in the case of benzodiazepines, very high doses of benzodiazepine administered over several days may be required without fully achieving symptom control, causing distress for patient and staff as well as a potentially fatal outcome. The exact contribution of activity of the high-affinity *α* *β* *δ*-GABA_A_-receptor site of GHB still remains elusive in the withdrawal process.

The administration of related compounds such as the use of benzodiazepine in alcohol withdrawal indicates that the administration of GHB could be a superior management approach reducing distress, length of hospitalization, and health care costs. Even further, similar to methadone maintenance, the controlled administration of GHB could be a future perspective for these patients.

In summary, in this case, self-administration of GBL represented the superior management approach for GHB-dependence, GHB-withdrawal, and delirium exceeding the effectiveness of benzodiazepine administered orally or intravenously. Although the administration of GBL—which is illegal in most legislatures—cannot be a routine approach, the administration of GHB, the metabolite of GBL, represents a viable option. The administration of GHB could be the future approach for the management of GHB-dependence, GHB-withdrawal, and GHB-induced delirium. The implications on length of stay and health care cost have not yet been assessed and merited further investigation.
